# A Bill for the Better Regulation of Medical Practice Throughout the United Kingdom

**Published:** 1845-01

**Authors:** 


### Art. XIX.

1. *A Bill for the better Regulation of Medical Practice throughout the United Kingdom. Prepared and brought in by Sir James Graham. Ordered to be printed, 7th August,* 1844. Folio.
2. *Charter of the Royal College of Surgeons of England, granted Sept. 14th,* 1834.—*Bye-laws and Ordinances of the Royal College of Surgeons of England.* 1844. (*Not published.*)
3. *A Statement of the Society of Apothecaries, on the subject of their Administration of the Apothecaries' Act.—An Address of the Society of Apothecaries to the General Practitioners of England and Wales, on the Provisions of the Bill "For the Better Regulation," &c.—London,* 1844. 8vo, pp. 44 and 56.
4. *A Manifesto by the Medical and Surgical Association of the Borough of Marylebone.—London,* 1844. 8vo, pp. 16.
5. *An Exposition of the Laws which relate to the Medical Profession in England, &c.*—By John Davies, m.d. &c.—*London,* 1844. 8vo.
6. *Remarks on Medical Reform, in two Letters addressed to Sir James Graham.* By Sir James Clark, Bart. m.d., f.r.s.—*London,* 1842-3. 8vo, pp. 30 and 40.
7. *An Introductory Discourse on the Duties and Conduct of Medical Students, &c.* By Sir B. C. Brodie, Bart.—*Lond.* 1843. 8vo, pp. 34.
8. *A Letter addressed to the Secretary of the North of England Medical Association.* By C. T. Carter, Esq. (*Not published.*) 1844.
9. *A Speech at the Derby Meeting of the Provincial Medical Association, &c.* By Charles Hastings, m.d. f.g.s. 1844. 8vo, pp. 28.

The momentous subject of Medical Reform, now irresistibly forcing itself on the attention of every member of the profession, seems naturally to present itself for examination under the following heads:

I. What are the Grievances complained of by the members of the Medical Profession, and for which they are seeking redress under the name of *Medical Reform?*

II. Are the Grievances of such a kind as to justify the cry for Reform so generally and so loudly raised?

III. Are the Grievances susceptible of redress through the means of Medical Reform, or through any other means?

IV. If the Grievances are susceptible of redress, are the means proposed in the Bill of Sir James Graham, or in the various publications on Medical Reform, calculated to yield it?

In an inquiry like the present, addressed to the members of the medical profession, it would be a work of supererogation, to enter into any detail respecting the nature of their grievances. It is not, therefore, our purpose, on this occasion, to offer any reply to the first two of these questions.

To the third we shall content ourselves with returning a simple but qualified affirmative. In the long catalogue of real grievances there are, certainly, some which no legal interference can remove; and there are some things paraded as grievances, which we would demur to admit as such. But we cannot enter into any examination of either of these points at present: they will, however, be incidentally noticed in the course of our inquiry.

To the last of these questions the present article is to be considered as a detailed reply. In drawing it up, we shall have to pass in review and submit to strict and, as we believe, impartial criticism, every proposition in the Bill, having any important relation to the medical profession. We shall, at the same time, take occasion to consider several other analogous matters of importance, which, although not noticed, or but slightly noticed in the Bill, have been justly regarded as evils calling loudly for reform. Our inquiry will thus come to embrace nearly the whole system of medical polity; including the entire subject of the Organization of the Profession, past, present, and prospective—at least in England. This will be seen by the following Outline of the chief subjects treated of; which we the more willingly introduce here, as the glimpse thereby afforded of the whole inquiry may, by showing beforehand the bearings of the different parts, facilitate the reader's appreciation of each, in the progress of perusal.

I. Education of Medical Practitioners.
II. Examination of Medical Practitioners.
III. Civil Equality of Medical Practitioners.
IV. Registration and Licensing of Medical Practitioners.
V. Incorporation of the Medical Profession.
VI. Representation of the Medical Profession.
VII. Government of the Medical Profession.
VIII. Privileges and Protection of the Medical Profession.

### I. *Of the Bill in relation to the Education of Medical Men.*

The essential thing, in regard to the education of medical men, is that it should be *good:* that is, that it should be sufficient, first, to cultivate and improve the intellect, so as to render it capable of receiving and profiting by scientific truths; and, secondly, convey a sufficient amount of medical knowledge to afford a fair warrant that the possessor is qualified to practice medicine, with probable benefit, at least without injury, to the public. In other words, care must be taken that the candidates for medical practice should have obtained—first, a good *general*, or what has been called *preliminary* education; and, secondly, a good *medical* education, before they are licensed to practise any department of the medical art. Now, it must be admitted that Sir James Graham's Bill either provides for directly, or has the means and power of providing for both of these essential requisites. And it further provides, that this education shall be equally good in every part of the empire.

Of course, in making these statements, it is taken for granted that the new body instituted for the government of the profession, the Council of Health and Medical Education, will be so constituted as to be well qualified to judge what is the fitting education for a medical practitioner, and also shall possess the desire to see such an education carried into effect. And when we consider that this is one of the main objects for which the council is to be established, it seems absurd to conceive that its members, however appointed, should not possess the qualifications and the disposition here assumed. We shall have afterwards to consider in what precise manner this council ought to be formed: we at present assume that it is a body both able and willing to perform its duties.

Nor is this the place to inquire into the expediency or advantage of establishing, more markedly than before, as is done by the Bill, different grades in the profession. Regarding these grades as established, we shall consider education, as provided by the Bill, in relation to each of them.

1. *Licentiates of medicine and surgery, or General Practitioners.* Nothing is fixed in the Bill with regard to the period of study, or course of education for this class of practitioners. One very important point, however, having essential relations to a good education, is determined by it, viz. the abolition of apprenticeships. The necessity of serving an apprenticeship interfered, most fatally, with the obtaining of a classical or general education; and the system was otherwise most injurious to the mind of the medical student, by fostering routine and empirical habits of thought and practice, obstructive to the after-reception of knowledge on more scientific and philosophical principles. A large portion of the period formerly devoted to the duties of the apprenticeship may be, and doubtless will be devoted to the acquisition of classical and general knowledge, and yet ample space will be left for the acquisition of medical knowledge.

The age at which the license is attainable is fixed at twenty-one; but it is a question well worth considering, whether this might not be advantageously raised to twenty-two or even to twenty-three. It may, indeed, be argued, (1) that, inasmuch as it is only in actual practice that the niceties of practical tact and skill can be acquired, and, (2) as the great majority of young men, on first entering the profession, will be more or less subordinate to their seniors, as assistants or otherwise,—there is no substantial reason for prolonging the period of probation beyond that fixed in the Bill. These reasons are not satisfactory to us; and we hope the point will be well weighed during the reconsideration of the Bill in committee. The following remarks on this point, by Sir James Clark, are most apposite. They probably indicate the best practical course:

"A youth possessed of the natural talents which qualify him for becoming a medical practitioner may easily acquire the requisite preliminary knowledge, and much more, if his education be well directed, by the time he has reached the eighteenth or nineteenth year of his age. He has still four years to devote exclusively to the study of his profession; for I agree with a late writer on Medical Reform [Sir B. Brodie] that young men are not generally fit to enter on the practice of their profession before twenty-three years of age. At the same time, I admit that fixing a particular age may often be attended with great inconvenience and sometimes injustice to candidates: if, therefore, an age is fixed, it ought, I think, to be a minimum. If a good general education is enforced *before* the student enters on his medical studies, and if his practical knowledge, as well as his scientific acquirements in medicine, are well tested, the age need form no impediment to the candidate's obtaining a license. When a young man has proved himself to possess a competent knowledge of his profession, he may fairly claim the privilege of exercising it."*

The Council of Health is fully authorized to demand, and doubtless will demand, that this period set apart for the acquisition of general and professional knowledge, shall be really so devoted.

In the first place, by enforcing a registry of students at all the medical schools, it has the means of ascertaining whether the students, previously to entering, possess the necessary amount of preliminary knowledge; and, subsequently, whether the prescribed courses of medical instruction have been really followed.

Secondly, the Council, having power to make such changes as it pleases, in any of the schemes of the course of study required by the colleges empowered to examine candidates for the license (§ 19); also to send its secretary, or any of its members, to attend at such examinations, and see that they are satisfactory, and, if they are found unsatisfactory, "to refuse to register upon the testimonials of the body so in default" (§ 25); and having, moreover, the power of giving or refusing their sanction to all the bye-laws of the colleges—including, of course, those relating to education (§ 22); it is clear that it can enforce such courses of study as it thinks proper. And, as already observed, it would be monstrous to suppose that a Council of Medical Education should sanction aught less than such a course of education—preliminary and professional—as the present state of medical science, and the high character and standing of its professors indicate as proper. It would, however, have been satisfactory to the friends of medical education—the best friends of the profession—if the Bill had contained some more positive enactments as to the period and course of study required of candidates for the license, as in the case of physicians and surgeons. It would also have been agreeable to observe some more efficient arrangement for testing, in all cases, the proficiency of the candidates in classical and general knowledge. If such an arrangement is not introduced into the Bill, we trust, at least, that care will be taken that the College of Health is kept sufficiently awake to its immense importance. For our own parts, we entirely coincide in opinion with Sir B. Brodie and Sir James Clark, in regarding the enforcement of a proper PRELIMINARY EDUCATION, as of equal importance, at least, with that of the strictly professional part of study. Here it was, as already observed, that the system of apprenticeships produced such disastrous effects,—throwing, in innumerable instances, a blight over the whole future intellectual progress of the individual. In reference to this fact, Sir Benjamin Brodie may well say, that "it is very much to the discredit of the great medical institutions in this country, that, except in some few instances, they have not given even an indirect encouragement to the obtaining a good general education, and, in *one instance*, the legislature have actually done their best to throw an impediment in the way."† In Sir James Clark's 'Second

* Second Letter to Sir James Graham, pp. 16-17.

† Introductory Discourse, p. 19. But while thus strongly condemning the system of apprenticeship which has hitherto prevailed, we wish to guard ourselves against being supposed to condemn *every* system of apprenticeship. On the contrary, we have an equally strong opinion, that no man can be qualified to enter upon practice for himself, who has *not* served a practical clinical training of some sort, under this or some other

Letter,' the reader will find this question treated of in an admirable manner. We can only find room for one brief extract here, but we earnestly recommend the perusal of the whole section, as a masterly exposition of this all-important subject:

"Had the scientific education of medical men been better attended to, the nation might have been spared the loss of much human life, and the fruitless expenditure of much treasure; and the public health might have attained a much higher standard than it has yet reached. It is from being uninstructed in the common principles of philosophy, and, consequently, unacquainted with the laws by which the various physical agents amidst which we live are regulated, and the effects of these in promoting health and inducing disease, that medical men have failed in some of their highest duties—that they have been less efficient ministers of health and less successful investigators of disease than they would otherwise have proved. It is in the power, as it is unquestionably the duty, of the legislature to put an end to such a state of things; and an excellent occasion now presents itself for improving the education of medical men generally, and, above all, in its most neglected, but not least important department—PRELIMINARY INSTRUCTION." (pp. 17-18.)

We shall hereafter have to consider the merits of the arrangements for examination generally, provided by the Bill. At present we shall only observe, that though we regard them as very defective, and think that they ought to be changed, we cannot bring ourselves to believe that they can fail to be tolerably effective in practice. We are well aware of the fears expressed by some, that the examining bodies, (the Colleges of Physicians and Surgeons,) may be led to sacrifice the interests of the class of licentiates to the interests of their own grades of the profession, by *keeping the former down* through the instrumentality of a low and lax system of examination. To say nothing of the monstrous professional treachery involved in such a project, we may surely hope that if it could be entertained, it would be defeated by the circumstance that the acts of the council of health will be all subject to the direct supervision of parliament, and must be performed under the unsleeping eye of public jealousy.

2. *Surgeons.* By making the minimum age at which the diploma or license of surgeon can be obtained, *twenty-five*, it is of course intended that the additional period of four years beyond that period at which the degree of licentiate may be obtained, shall be devoted to study in schools and hospitals. It cannot, therefore, be doubted, that if the licentiate were at all qualified for the practice of his profession by his course of study, the surgeon must be still better qualified generally, and especially in what may be regarded as his peculiar department, viz. the treatment of external diseases, and chirurgical operations. While admitting this, we name. A dressership, a clinical clerkship, a house-surgeoncy in an hospital, are all apprenticeships, and universally acknowledged to be of the last importance to the students who are fortunate enough to obtain them. But the great majority of general practitioners cannot expect to hold such offices; and it seems, therefore, essential that some equivalent should be found in their case. At present, we do not feel ourselves called on to discuss the subject, but we claim for it the earnest attention of all the friends of medical education at the present crisis. The objections to even an ordinary apprenticeship (of one or two years) do not apply, with anything like the same force, *after* the student has obtained a good preliminary education, as before he has obtained this. But if this should be thought objectionable or unattainable, might it not be a regulation that men *licensed* at the age of 21 should not be allowed to practise *independently*, but only as ASSISTANTS of other practitioners, until they had reached the age of 22 or 23?

cannot overlook the fact, that, as far as the test of *qualification by examination* goes, the licentiate may be better qualified, in one most important department, than the surgeon, viz. in *practical medicine.* By the enactments of the Bill, the licentiate is required to pass examinations in every department of medical practice. In England, for example, he must be examined "by the Royal College of Physicians of England, assisted by the Court of Examiners of the Apothecaries' Company; and also by the Royal College of Surgeons of England," (§ 14.) Of course, the object of sending the licentiate before the College of Physicians, is to test his knowledge of practical medicine, the practice of which, it need not be stated, constitutes by much the greater portion of his professional duties. The surgeon, however, is only required to be "examined by one of the Royal College of Surgeons of England, Scotland, or Ireland." (§ 15.) So that as far as practical medicine is concerned, no means is supplied by the Bill for testing the knowledge of the surgeon. In the new bye-laws of the College of Surgeons it is, no doubt, provided that the candidate for the fellowship shall have "attended the medical practice of a recognized hospital for one year, and lectures on the theory and practice of medicine and on clinical medicine, during two sessions of six months each;" and although it may be fairly presumed that the opportunities afforded by such a course of study as is prescribed, will, in reality, lead to the acquirement of a sufficient stock of medical as well as of surgical knowledge; it is certainly odd that, considering that more than half the practice of all pure surgeons is really medical, no positive provision should have been made by the Bill for testing such knowledge. We presume that this anomaly has arisen not intentionally, but from oversight, and that it will be remedied in the passage of the Bill through the House. The obvious amendment (on the basis of the present Bill) is, that the surgeon, as well as the licentiate, should be examined "by the College of Physicians, assisted by the Apothecaries' Company."

With the exception just stated, the education required by the Charter and Bye-laws of the College of Surgeons is most ample. Indeed, if any fault can be found with the curriculum, it is that it is too extensive. At any rate, no one who reads the Statutes respecting candidates for the Fellowship, as set forth in the "Bye-laws and Ordinances," can entertain any doubt that it will be henceforth impossible for any person to become a Fellow without being possessed of an ample stock of general and surgical knowledge, or that the Fellows, as a body, can be other than accomplished surgeons, in every sense of the word.

3. *Physicians.* The provisions of the Bill seem ample for securing a good and sufficient education for the physician, indeed (with one single exception) almost too ample. In the first place, he must have obtained his degree in some British University, at which he has resided and studied at least two years (§ 20;) secondly, he must have "applied himself to medical studies during at least five years" before he is allowed to take this degree (§ 16;) thirdly, he must have been *afterwards* "examined by one of the Royal Colleges of Physicians of England, Scotland, or Ireland," (§ 16,) and must have "received letters testimonial from the Examining College, of his being duly qualified to practise as a physician," (§ 16;) and lastly, he must have attained his twenty-sixth year before he is registered for practice. (§ 16.)

The few deviations from these regulations, enabling general practitioners to graduate after forty years of age, and enabling the graduates of foreign universities to be licensed as physicians, are hardly worth mentioning; and, at any rate, they do not, in any way invalidate the proposition here maintained, that, with the exception already hinted at, the qualifications of physicians are most fully provided for by the Bill. The exception here alluded to, is the non-enforcement of surgical studies and the want of any examination in surgery. Although an acquaintance with surgery is not so absolutely necessary to the physician as an acquaintance with practical medicine is to the surgeon—few physicians treating surgical diseases—still we hold it to be an axiom based on both reason and experience, that a knowledge of both departments—a *general* knowledge at least—is essential to the practitioner in either.

From this survey it seems to us to be satisfactorily made out, that, even under the defective arrangements for examination established by the Bill, a good though not a complete education will be enforced in all the grades of the profession; and it may be fairly presumed that, under its operation, no medical man will be henceforth licensed to practice who has not shown that he is qualified to do so. Of course, no Bill and no human arrangement can communicate, or can even test the existence of, much that it is most important that a medical practitioner should possess. The Bill cannot make a stupid man clever, or give the power of observation and logical inference to a mind that has it not; neither can it confer the capacity and inclination for continuous study and improvement, or bestow habits of industry, attention, kindness, &c., so essential in the exercise of the medical profession. But it is here decidedly provided for, (always assuming the Council of Health to do its duty,) that every future practitioner must possess such an amount of medical knowledge as must be a satisfactory guarantee both to the members of his own profession and to the public, of his competency to treat disease.

### II. *Of the Bill, in relation to the Course of Study and Mode of Examination of Candidates for the License.*

Beside ensuring a good education in every grade of the profession, the Bill introduces a simplicity and uniformity in the modes whereby this is to be acquired and tested, which constitute decided improvements on the old system. In the first place, the Council of Health is empowered to take care that the same or similar courses of study shall be required throughout the three kingdoms, for each of the three grades into which the profession is divided. Secondly, the educational requirements being the same, the nature of the examinations, and also the amount of fees, will, of course, be the same, or as nearly the same as circumstances may permit, (§ 19.) Thirdly, the students may repair to whatever examination board may be most convenient or agreeable to themselves, e.g., Irish students to the London or Edinburgh board, or English and Scotch students to the Dublin board. Fourthly, the examination boards being reduced to one in England and Ireland, and two in Scotland, [the expediency of reducing these to one is most obvious,] and all constituted on the same general plan, there is introduced a facility of understanding, of planning, and directing the career and educational course of young men destined to the medical profession, which must be very satisfactory to their guardians and advisers, as well as to the students themselves.

So far, then, we must admit, that the provisions of the Bill are satisfactory as regards the prescribed modes of obtaining knowledge, and the *exterior economy* of the arrangements for testing the qualifications of candidates. The Bill, however, contains two great blemishes bearing on the *interior economy* of these arrangements,—one of commission, the other of omission.

1. The first of these blemishes is the proposed *Constitution of the Examining Boards*, which presents several obvious defects.

*a.* We have already referred to an objection founded on the supposed prejudice of the intended examining bodies (the Colleges,) against the class of licentiates. While demurring to the degree and influence of the prejudice implied in the objection, we cannot deny its existence altogether, and must therefore admit the possibility of its evil operation in the way supposed. We have given above some reasons for believing that if it did exist it would be rendered comparatively inoperative. Others, however, think differently; and it must be admitted that *the very belief of its existence* by the general practitioner, is in itself a serious evil, and affords an argument of no mean weight against the constitution of the boards as proposed by the Bill. This objection has been strongly urged in many quarters, and is thus forcibly stated in the Marylebone Manifesto and in the Apothecaries' Address:

"The provision by which the education of the future general practitioner is placed under the control of a mixed board of physicians, surgeons, and apothecaries, is bad in principle. In such a board the physicians and surgeons will always possess a preponderating influence over the apothecaries. They will be in the dilemma of either continuing the general practitioners upon an equal footing with themselves, in point of medical and surgical education, or of constituting them an inferior grade. The interest of the class represented by the more influential part of such a board, namely, the consulting practitioners in medicine and surgery, will always be to keep a lower grade at as great a distance from their own, in point of medical qualifications, as possible. Thus, even if a penal clause to prevent unlicensed practice were appended to the Bill, the educational tendency of such a board, as respects the great mass of medical men, under whose care the health, limbs, and lives of the majority of the people must fall, would be in the wrong direction." (Manifesto, p. 9.)

"The interest of the physicians, as a body, is directly opposed to this improvement and elevation of the general practitioner. The difficulty which this view of the subject presents to the examination of the general practitioner by the physician, has been felt and acknowledged by members of that body, because it cannot fail to render the duty an invidious one, and there will always be this anomaly attending its discharge, that in proportion as the duty of giving a high qualification to the general practitioner is performed with zeal and efficiency, will the physicians be breaking down the distinction which exists between the class which they are educating and themselves, that distinction upon which their very existence, as a separate class, depends. Looking to the constitution of the governing body of the College of Surgeons, the same reasoning applies, and with equal force, to the share which that body is to take in the education of the general practitioner. But it will be said, you ought not to impute to the Colleges of Physicians and Surgeons, that in the exercise of the powers proposed to be given them by this Bill, they will be actuated by a desire to lower the standard of attainment of the general practitioners, or check their improvement as a class. We reply that our objection to the Bill is, that it gives them the *power* of doing so." (Address, p. 30.)

*b*. The partialities of corporate bodies in favour of their own members are notorious. In the present case, what guarantee have we that the Colleges will select for examiners the men best qualified for the office? We think the chances are much against their doing so. It is not the oldest physician or surgeon, nor yet the man of largest practice and greatest professional experience—the man of most influence in the College—that is likely to be the most competent examiner. The Colleges will, of course, restrict the selection to members of their own body, most likely to members of their Council; now it is a very possible case, that the men most qualified to examine may not belong to the College at all, or hold any prominent place in its ranks. We agree entirely with Sir James Clark in thinking, that "such bodies (the Colleges) are not qualified to test candidates on their scientific acquirements."*

"That power should be intrusted only to an independent body, unconnected with the educating institutions on the one hand, and the medical corporations on the other; a body responsible to the government for its acts, having no collateral interests to divert its attention from carying out in the fullest manner the principles embodied in the legislative enactment."†

*c*. Another most serious objection against the examination boards proposed, is one that has reference to the second of the great blemishes of this part of the Bill, which we shall consider directly. The licentiate of medicine and surgery will have no more reluctance to come before the examiners of the College of Physicians than before the examiners of the College of Surgeons. But the case is very different with the future members of the Colleges themselves. The expectant Associate of the College of Physicians, the B.M. or M.D. of an university of repute, will feel it to be somewhat *infra dignitatem*, to submit himself to the questioning of the Board of Surgeons, and even to the examiners of his own college *when "assisted by the court of examiners of the Apothecaries' Company*." The surgeons, on the other hand, will repudiate the examination of the physicians. Accordingly, the Colleges, more especially the College of Physicians, at least the members individually, do not, we believe, hesitate to point to this double constitution of the examining board, as a main reason for refusing to have their members examined by any other than their own section of it.

For these and for many other reasons which our limited space prevents our noticing, we entertain a very strong opinion that the Examination Board should be constituted independently of the Colleges, and that the members should be nominated by the Council of Health, to hold office for a limited period, but to be reeligible after its expiration.

2. The second defect, under this head, has just been referred to: it is

* No offence is meant in making this remark. It is founded on the conviction, derived from experience, that few men are qualified for the office of examiners who are not *teachers*—men obliged to keep up their knowledge to the level of the present day, and habituated to the practice of examining.

† Second Letter to Sir James Graham, p. 29.

this—that the Bill does not contain a clause to render it obligatory on every candidate for the license to practise, to undergo the same preliminary examination. In other words, we are of opinion, with Sir James Clark, "that every practitioner, whatever may be the department for which he is destined, should qualify for the duties of the general practitioner," and, in the first instance, submit to the same examination. We would even contend with the same writer, that this examination should be the only one requisite to qualify for practice in any of the grades of the profession; although the subsequent collegiate examinations might still be properly retained, and the possession of the titles flowing from such collegiate examinations, continue to confer peculiar privileges. Few men can now be found in the profession who will deny the essential unity of medicine in all its forms, or contend that the physician should not understand surgery or the surgeon physic. The one examination here proposed would ensure the possession of this general knowledge by all members of the medical body. The common examination, and the common title and license thence derived, could not fail to have a healing influence on the divisions, bickerings, and jealousies that now distract the profession at large, and which, it is to be feared, will be much increased instead of being diminished by the Bill, if carried in its present form. By the plan we are advocating, every one would, in the first instance, be licensed under the common title of "Licentiate of Medicine and Surgery;" but, of course, no one would be debarred from proceeding to seek other titles given under other conditions, and conferring other privileges; or be prevented from devoting himself to whatever department of practice he preferred.

### III. *Of the Bill, in relation to the Equality of Privileges of Medical Men in Practice.*

Here the Bill confers everything which the profession has sought for or can reasonably desire, viz. perfect equality of rights and privileges to every one of the same grade throughout the empire. No college or corporation, nor any other public body, can henceforth interfere with the practice of any individual, in any part of the three kingdoms, wheresoever he may have studied or may have obtained his license to practise. As the Bill, in the enactments last considered, establishes *uniformity of education and qualification among practitioners of the same class, throughout the empire;* so, in that now under review, it establishes *uniformity of privileges*, in the same degree. All the limitation introduced, in respect to community of privileges throughout the empire, is, that physicians and surgeons removing from the part of the kingdom where they have received their letters testimonial, to another, for the purpose of practising, must enrol themselves as fellows or associates of the college of the kingdom to which they remove, paying the usual fees, but without undergoing any examination. (§ 18.) No restriction of this or any other sort applies to the licentiates; and the reason for its being applied to the two other classes is not very obvious.

### IV. *Of the Bill, in relation to the Registration of Licensed Practitioners.*

The plan of a general registration of practitioners, by the Council of Health, and the official publication of a register, for the information of the profession and the public, is an immense improvement on the old system, and cannot fail to be of great benefit. The plan, as proposed in the Bill, is as follows: Every practitioner, after receiving his testimonials from the examining boards, may, on the payment of a fee, have his name inserted in a general register, kept by the Council of Health. This register is to be " published from time to time" [annually]. " And every person whose name shall be so registered, who shall be desirous that his name shall be continued in the published register, shall, in the month of January, in every year," renew his application for registry, when the Council " shall forthwith cause the names of all persons so returned to them, to be published in alphabetical order, in their several classes, with their several places of abode, and dates of their testimonials." (§ 13.)

The establishment of a general registration of all legally-qualified practitioners, and the annual publication of an authentic register of their names, is a measure calculated to be of such essential service to the profession and the public, that it cannot be too cordially received; and no pains should be spared to make this part of the scheme as perfect and practical, and as easily-worked as possible.* Some objections have been made to the plan as stated in the Bill; and some alterations, with a view to its improvement, have been suggested, which merit examination.

1. In the first place, it is objected that the registration is not made *compulsory* in every case. But it is so made to all who wish to be considered as legally-qualified practitioners. In fact, so far as appears by the Bill, this act of registration seems identical with, or to form a part of, the act of licensing. At any rate, it is quite clear that it ought to be made compulsory for every one to register, in the first instance, at least, if it is not already so provided by the bill.

2. Some have objected to the clause rendering it obligatory on every one to send " to the Council his name and place of abode" every year; on account of the trouble, especially where there is no change of abode. But the trouble imposed is insignificant, compared with the attainment of the great object of having a correct register. It is much less trouble than returning the names of ourselves and servants annually to the tax-gatherer: still even this ought to be avoided, if the plan can be well worked without it.

3. Much stronger objections have been made to the amount of the

* One of the many obvious and important benefits to be derived from an authentic public register, was noticed by Mr. Swain of Devonport, at one of the late meetings. Every one who has resided in the country must have witnessed similar cases to those adverted to by Mr. Swain. " They could all of them," he said, " speak of the annoyance of persons coming among them and practising under the plea that they were qualified to do so; but great difficulty at present existed to ascertain whether they were so qualified or otherwise. They met with persons practising as medical men, whom nobody knew anything about; and although they might ask who they were, they could not always hope for a satisfactory answer to their inquiries." (Lancet, Oct. 12.) Of course, a single glance at ' The Register,' if it existed, would cure this evil.

registration-fee, viz. £5 in the case of a physician or surgeon, and £2 in the case of a licentiate. But if we are right in thinking that this fee is not merely for the registration, but is also for the license to practise, it will seem less exorbitant. Still, as no obstacle that can be avoided, should be thrown in the way of registration, it is highly desirable that the registration-fee should be made as small as the "defraying of the expenses of this act" shall justify. The estimation of the just amount of fee will depend partly on the kind and amount of privileges accruing to the registered. For instance, in what form is the register to be published? Is a fresh register to be published every year? Is a separate copy of it to be delivered gratis to each of the registered, or is it to be sold? If sold, is the price to be high or low?

But here, as in many other parts of the Bill, the appreciation of the particular enactment is rendered abortive by the knowledge that the Bill contains one monstrous clause, which has an important bearing on every other, and which clause, we feel confident, must and will be entirely changed before the passing of the Act. We, of course, refer to that part of the Bill which leaves the practice of medicine totally unrestricted. If this clause were altered, and it were part of the enactment that the licensed alone were privileged to practise the medical art, there would then no longer remain any doubt that the registration should be made compulsory, and there would be then little reason for complaining of the amount of the fee, for securing a privilege of such real importance.

V. *Of the Bill, in relation to the Incorporation of the Profession.*

The Bill makes no direct attempt to incorporate the medical profession. By registering the members, however, it formally recognizes them, for the first time, as a distinct body; and by placing the Council of Health at their head, connects them, in some sort, with the state. This, as formerly remarked, is an important improvement; but it is not the kind of incorporation which reformers have desiderated. What they have contended for, as a matter of right flowing from their very qualification as practitioners, is enrolment as members of some college or institution, in which they might feel themselves, as it were, at home in their own hall, and have a right to take a part, more or less, in the discussion and direction of their own affairs. If the Bill had contained any arrangement by which such an extensive change could have been effected, it would, unquestionably, have been a boon of great value to the profession, more especially to the largest and most influential part of it, the general practitioners. But the difficulties attending any such incorporation were, doubtless, regarded by the framers of this Bill as insurmountable; or, at least, as involving such opposition from the existing corporations, as would have rendered the passing of anything like a good bill most difficult, if not impossible. The changes made are, therefore, partial and inconsiderable. This is a great misfortune; because nothing would tend so much to produce harmony, good will, and content in the profession, as the union of the different classes in one great college or corporation; or into two or more colleges, separated according to pursuits or grades, yet joined together in a sort of federal union. It is always dangerous to leave any large body of men, of the same general class, to see and feel that they are separated from their brethren by any impassable line of distinction, indicating superiority and inferiority, real, nominal, or conventional. But we believe that the defects of the Bill in this respect, are attributable not so much to its framers as to the members of the profession themselves. It is the old medical corporations, the Colleges of Physicians and Surgeons, that have always stood in the way of such an incorporation of the members of the profession, as would be generally satisfactory: and it is understood to be to their opposition, on the present occasion, that we are indebted for the deficiencies in the Bill which we are now considering. The mistake committed by the minister—and it is a great mistake—was to grant any of the new Charters previously to the passing of the Bill.

It is, however, but just to the colleges and to the government, to admit, that, as far as set forth in the Bill, and, as far as we know, in the charters already granted, or about to be granted to the former, the principle of incorporation is carried a good deal further than it was before. A survey of the different classes of practitioners, in regard to corporate privileges, will show this clearly enough; but it will also show how very inadequate the amount of incorporation still is, to meet the wants of the profession at large.

### I. PHYSICIANS.

It appears by the Bill, that every physician, on obtaining his letters testimonial, and being registered, will be enrolled as an *Associate* of some Royal College. He will thus have a name and station at least; and all that could be reasonably asked for further is simply, (1) that, in his capacity of associate, he should have the right and privilege to meet with his brother associates in the halls of the college, to consider and discuss such affairs as concern the body to which he belongs; and (2) that he shall eventually attain, or at least may attain, the higher dignity of *Fellow*. Whether the process whereby this elevation is effected be by simple seniority or by examination, does not seem material, as long as the dignity is *equally open to all*. But this last is essential. It is, indeed, said that by the terms of the new charter prepared for the College, the number of Fellows is to be limited, and that the limited number is to be created by arbitrary selection and election by the Fellows and Associates. But we can hardly believe that a rule at once so unjust, and impolitic, and one which, we cannot but think, must be obnoxious to the feelings both of the Fellows and Associates, can be sanctioned by the present Fellows of the College. Such a plan must inevitably excite dissatisfaction and discontent among the great body of the Associates, and instead of attachment to the College, must lead at least to estrangement if not to open hostility. The poor Licentiate, Heaven knows, has already been long enough an outcast; and surely it is not too much to expect better treatment for him under his new title of Associate. Wherefore, —knowing, as we do, their general high character and liberal feelings as individuals,—we confidently appeal to the Fellows, in the Hall of their College, and in their corporate capacity, to do him justice.—"Quamobrem magna me spes tenet, si ea, quæ sunt in causa, explicare, atque omnia dicendo, consequi potuero, hunc locum consessumque vestrum, quem Illi horribilem ac formidolosum fore putaverunt, eum tandem ejus fortunæ miseræ multumque jactatæ, portum ac refugium futurum."*

The mode of admission to the Fellowship, adopted by the College of Surgeons, that, viz. by examination, is much fairer than the one just referred to, because it leaves the door open to every one who may choose to fulfil the conditions requisite to enable him to pass through it. The plan by examination, however, could hardly be adopted in the College of Physicians, because the studies and examinations undergone for the Associateship are already fixed so high. The only remaining plan, and certainly the best of all, (if the two classes of physicians are to be maintained,) is that by seniority, every Associate of a certain standing—say seven or ten years—claiming a right to admission to the Fellowship on attaining the fixed probationary limit, unless some tangible objection can be proved against him.

The following occur to us as the only objections of any force that can be urged against this plan:

1st. That an honour shared by so many, and alike open to all, men of talent and men of no talent, would cease to be regarded as an honour. Some force might be allowed to this argument if the practice which it purposes to supersede, was calculated to promote the honoring of desert alone, or was even likely to do so at all. But as we have no guarantee in past experience that this will be the case in future; and as, indeed, there is every reason to believe such would not be the case; we are bound to maintain that the argument derived from the obvious manifold evils necessarily flowing from the practice of *selection*, is all-powerful. But it is part of the plan advocated by us to leave a door open for the promotion of distinguished merit, even under the system of seniority. We admit that it would be a defect in the arrangement if there were no means of enabling the College to testify its sense of the value of great discoveries or of other important services rendered to science or humanity by its Associates: and we would therefore leave the power in the hands of the President and Council to confer the fellowship on a certain number—say two or three annually—who could not claim it on the ground of seniority.

2d. It may be urged, in the second place, that, according to the system of seniority, persons of disreputable moral character might occasionally find entrance to the fellowship. This danger, however, could be easily obviated in various ways. For instance, it might be arranged that in addition to the claim of seniority, every associate should be required to produce a certificate or recommendation, signed by a certain number of fellows, (as in the case of the Fellowship of the College of Surgeons,) to the effect that he is "a fit and proper person to be admitted to the fellowship." Or even a ballot might be instituted for every one as he attained the limit of seniority, the number of excluding balls being fixed at such a high proportion (say two thirds) as would prevent the effective operation of any mere prejudice or spite, and yet would effectually obstruct the admission of persons justly obnoxious.

3d. It may be urged that so very large a body as would be constituted by all the associates becoming fellows in their turn, would form a very bad medium for the transaction of business. To this we reply that, for the mere *discussion* of the affairs of any body of men, a large assembly is by no means either improper or inconvenient; and that for the *transaction*

* Cic. Orat. pro A. Cluent.

of what is more properly termed *business*, the College has the same medium as other large bodies, viz. its Council. It would be an easy matter to decide what subjects, whether for deliberation or execution, should be intrusted to the Council; and no one could object to all the *business* being transacted by it, so long as its members were chosen by the Fellows at large. In order to ensure a fitting executive of this kind, and, at the same time, to accord to seniority and experience their just due, all would agree to its being made a law of the corporation, that the members of the Council should be elected from Fellows of a certain standing, and the President from a still smaller number at the head of the list.

A College, constituted as is here proposed, would present an example of a real incorporation, and a just representation of one department of the profession; and while we see how easily, and at how little sacrifice the change could be effected, we cannot bring ourselves to believe that the College of Physicians will persist in what is understood to be their intention respecting the admission to the Fellowship.

### II. SURGEONS.

In treating of this class of the profession, our views are not restricted, as in the case of the physicians, by imperfect documents. We can here refer not merely to the general Bill but to the Charter and the Bye-laws of the particular College, all throwing light on each other, and together constituting the whole documentary evidence likely for some time to exist on the subject. From these we gather the following particulars,—few, indeed, but all that have reference to the subject of incorporation:

The College consists of two classes, MEMBERS and FELLOWS, both unlimited as to number, but differing materially as to rights and privileges.

MEMBERS. The documents give us but little information respecting members; they are, however, spoken of, both in the Charter and Bye-laws, as one of the constituent classes of the College. Nothing is said respecting the mode of their admission to the Collegiate honour, nor of the general privileges attaching to the membership. As, however, the new Charter confirms "all jurisdiction, powers, authorities, &c. as to the admission and expulsion of members," formerly possessed by the College, it is to be presumed that the regulations as to the attainment of the diploma, and also the common privileges thereby conferred, will be the same or similar to those now in force. It is generally understood—and we ourselves firmly believe—that that portion of the examination for the *Licentiateship*, which is to take place before the College of Surgeons, will be regarded as identical with that for the membership, the diploma being immediately thereafter attainable on the payment of a certain fee. No hint is given in the documents as to any corporate privileges attaching to the membership, except the very important one of its giving a claim to examination for the Fellowship, a certain number of years after the date of the diploma. The value of this privilege will be understood when we come to consider the privileges of Fellows. The future members will, of course, retain all the rights and privileges, such as they are, possessed by the present—such as free access to the library, museum, and theatre of the College, &c.

FELLOWS. According to the provisions of the Bill, no person can be registered as a "surgeon" unless he have attained the age of twenty-five, have applied himself to surgical studies during at least five years, and have passed an examination before one of the Royal Colleges of Surgeons. On being registered, he becomes, *ipso facto*, a Fellow of the College.

According to the Bye-laws, there are two ways of admission to the Fellowship, the one having no necessary connexion with previous membership or professional practice, the other having a necessary connexion with both.

1. By the first, any person who is twenty-five years of age; who is recommended by three Fellows as "a fit and proper person to be admitted to the Fellowship;" who can produce proofs that he has studied during certain prescribed terms at schools and hospitals, (a most ample curriculum;) "that he has attained a competent knowledge of the Greek, Latin, and French languages, and of the elements of mathematics"; "that he has served the office of House-surgeon or Dresser in a recognized hospital in the United Kingdom;" may present himself for examination for the Fellowship, and if he "shall be found qualified for the same," he shall receive his "Diploma of Fellowship." Candidates of this class may, or may not, have previously become "members" of the College.

2. The second medium of admission to the Fellowship is through the previous qualification as Member. It is recited in Clause 7, Sect. I, of the Bye-laws, "that any member shall, after the expiration of twelve years from the date of his diploma,* be admissible to examination for the Fellowship, upon the production of a certificate signed by three Fellows, to the effect, "that he has been twelve years in the practice of the profession of surgery; that he is a fit and proper person to be admitted a Fellow, if upon examination he shall be found qualified;" and also, "if he have not the degree of Bachelor of Arts, that he has a competent knowledge of the Greek, Latin, and French languages, and of the Elements of Mathematics." Upon passing the examination, as in the former case, the candidate will receive the Diploma of Fellowship, and be registered as a Surgeon.

As we have no means of knowing what curriculum of study may be required of candidates for the *membership* in the first instance, we cannot say to what precise extent this second mode of admission to the Fellowship is a special boon to the Member in regard to school or hospital attendance; but we take it for granted that the "twelve years' practice" is intended to stand as an equivalent for the "Dressership," and for certain other stringent conditions as to study, required of the junior class of candidates. We should also expect, as a reasonable concession to the acknowledged influence of time in dulling the vivacity of scholastic impressions, that the examination of this class of persons would be less stringent in matters of mere science, and more practical, than that of the other class.

The rights—certainly and probably—attaching to the Fellowship, show a considerable advance in the general views respecting medical incorporation, as well as an important augmentation of the dignity and professional privileges of the particular class of surgeons who are to be possessors of it. For the first time, since the institution of the College, a

* We notice this as the general rule. An exception is made in favour of those who have been members at the time of granting the Charter; they may go up for examination at the expiration of eight years.

considerable body of its members,* under the name of Fellows, are entitled to meet in the Hall of the College, to elect their executive body and their own officers, by free ballot. They are themselves also eligible to fill any of the offices of the Corporation, under certain conditions.† It is to be presumed, also, that there exist the means of calling other meetings beside those for the election of the Council, at which Fellows may have an opportunity of discussing the affairs of the College or of the class of the profession to which they belong.

As far, then, as regards the class of Surgeons called Fellows, there seems little to desire in the way of incorporation beyond what is conceded by the Bill and by the Charter; and if the whole profession consisted of two classes only, Physicians and Surgeons (in the restricted sense,) we should, for our own parts, after certain easy modifications of their bye-laws, be well enough contented with the organization of the Colleges. But it is impossible to consider this subject without going further, and inquiring how the interests of the largest and most important class of all, the General Practitioners, stand affected by the two forms of incorporation which we have been considering.

### III. GENERAL PRACTITIONERS.

It is of no practical use to investigate the causes of the division of the profession in this country into three classes, or to reason on its expediency or inexpediency; it suffices that it exists and must continue to exist. The only questions of real importance to be now considered are, (1) Whether the general interests, the honour, the happiness of the general practitioners, as a constituent body in the profession, are sufficiently conciliated by the arrangements of the established Corporations; and (2) If they should appear not to be so, how such a conciliation is to be effected.

No one will attempt to dispute the right of this body—forming nine-tenths at least of the whole profession—to have its interests considered, weighed, and adjusted. It may, indeed, be argued, whether that association and organization of its members, usually understood by the term Incorporation, is really essential to the best interests of the class of general practitioners. Much difference of opinion will exist on this point; but the difference, we apprehend, will be rather as to the meaning of terms and the amount of the thing sought for, than as to the expediency of the thing itself. It is contrary to all experience, and indeed in contradiction to the principles of human nature itself, that any large proportion of a body of men having such identical pursuits as the whole medical profession has, should sit down content under the privation of powers, privileges, honours, titles, (whatever they may be,) springing out of the very nature of these pursuits, while they see them exclusively attainable and exclusively enjoyed by a small fragment of their number. We say *exclusively attainable;* because men will reconcile themselves to any obstacle in the way of their ambition, provided they have the conviction that it is by possibility removeable, and free scope is left in the mind for the exercise of hope and the indulgence of rational expectation. And

* The present number of Fellows is upwards of 500.

† Some of the regulations respecting the election of the Council, as laid down in the Charter, are so manifestly bad, that they will require to be changed. Our business, at present, is to consider principles not details.

so it is with the general practitioners of England. Knowing themselves to be brothers and fellow-labourers in the same field with Physicians and Surgeons, they naturally expect that they should partake of the same fruits and enjoy the same or similar rewards,—at least in a degree bearing some proportion to the comparative dignity of their status and the value of their labours. If this expectation is not fulfilled, it is unreasonable to look for content among their ranks. That content is *not* found there at present, we have the testimony of the almost unanimous voice of this part of the profession, proclaiming aloud from one end of the kingdom to the other,—not merely that their actual condition must be changed, but that their prospective condition, shadowed forth in the Government Bill and the College Charters, is equally unsatisfactory.

One of their loudest calls is for *Incorporation*. Incorporation they see exists, and is prized in the other classes of their profession; it confers peculiar rights and privileges on the incorporated members; it matters not a whit whether these are of any real value, it is enough that they are the object of desire: if the path that leads to them is barred against a class that claims participation in them as a right, that class, as a matter of course, is discontented and unhappy. But the justest cry for justice may be unreasonably exalted by passion. Let us, therefore, while conceding to the general practitioner his undoubted right to share in the privileges of Incorporation, examine with calmness and impartiality his true position, present and prospective, in regard to this privilege, and then decide, according to the best of our judgment, how his wrongs, if found to exist, may be best redressed.

It is scarcely necessary to remark that the present anomalous position of the general practitioner in regard to incorporation, arises from the fact, that the medical profession, in this country, has finally resolved itself into three departments, while the corporations formed previously to the tripartite division, have still remained but two.*

The members of the new third estate of the medical kingdom, the general practitioners, combining as they do, in their own persons, the functions of the members of both corporations, might naturally seek alliance with either, in their desire for incorporation. But association with the physicians was, speaking generally, impossible, from the want of an University degree. They therefore joined the incorporation of surgeons, and, under the name of Members of the College, have continued, until the present time, associated with them, at least in name. But, as might be expected, this union, legally voluntary yet conventionally compulsory, proved anything but satisfactory to the general practitioners; and their long discontent and loud claims for something more than a mere nominal alliance, have mainly brought about the state of

* In making this statement we purposely diregard the Apothecaries' Company, because it has no claim whatever to be considered as a medium of incorporation for the general practitioner. It had none of the fundamental principles requisite to entitle it to be so considered, and it never was so considered by its members.

The sudden cry recently raised in favour of this body, must have surprised the Worshipful Company fully as much as it has surprised everybody else. And yet the cry was natural, though strangely misdirected. The just terror of the impending blow from Sir James's non-restriction clause has somewhat disturbed the judgment of the general practitioner. To escape from the Quacks he would take refuge in Apothecaries' Hall, just as a man pursued by a mad bull would jump into a horsepond. But surely neither the one nor the other would select such a resting-place in cool blood.

things leading to the changes in the medical constitution, effected or projected, now under consideration.

According to our representation of the privileges of the Associates and Fellows of the Colleges of Physicians and Surgeons, it cannot be denied that both these societies afford, or may easily be brought to afford, tolerably fair examples of incorporation, as far as regards their own members. And as they are composed of men avowedly belonging to those orders of the profession most regarded by the world, affiliation with their ranks ought to be generally satisfactory. Doubtless it would be so to the general practitioners, provided it could be attained on anything like equal terms, or on terms not derogatory to their standing as gentlemen and men of education; and provided it presented the essential quality of being *attainable by all.* In theory, no doubt, this is the case; but many of the conditions are of such a nature that though it may be *possible* for all to accept them, it is more than improbable that all will ever do so.

Although we have already stated these conditions we will repeat them here; and we will also repeat our opinion that, however inadequate they may be considered by general practitioners, they are, in truth, boons to their order in the way of incorporation, altogether new, and the only substantial boons in this respect they have ever received.

1. All persons having passed as licentiates of medicine and surgery, may immediately become members of the College of Surgeons on paying a small fee for the diploma. They will then possess every privilege in respect to the College which they now possess. They will be MEMBERS OF THE ROYAL COLLEGE OF SURGEONS, and may put "SURGEON" on their cards and on their doors as heretofore, although they cannot appear in the Register under this title.* Twelve years after the date of their diploma as member, they may claim to be examined for the Fellowship, and on passing the examination and paying the fees, will be registered as "SURGEON," and be constituted FELLOWS OF THE COLLEGE OF SURGEONS with all the privileges. Now, if the examination is such a one as may reasonably be expected to be passed by men who have been twelve years absent from the schools, but who have not foregone their private studies, it cannot be maintained that the access to corporate privileges is excessively and unreasonably difficult. According to the Bill, the Fellowship may be thus attained at the age of 33, a period of life which cannot be regarded as unreasonably advanced, for a man to wait for, ere he can acquire official distinction. If we could believe that the arrangement would be regarded generally in this point of view, and that all or even a large majority of the Licentiates of medicine and surgery, would become members with the purpose of eventually becoming Fellows, we should feel that a good deal was done towards effecting a tolerable incorporation of this class of the profession. The arrangement would, at least, produce a result of the greatest possible value to the public at large, that of ensuring a highly-educated and highly-qualified class of general practitioners. This is a result of such paramount importance, that no effort should be spared to bring it about. And we earnestly entreat the Council of the College of Surgeons to reconsider those of their bye-laws which have reference to the admission

* Persons who were members of the college at the time of passing the Bill, could claim to be registered as surgeons.

of Fellows, with a view to rendering them less stringent than they are. We are far from proposing that they should be rendered so lax as that any risk should be run of seriously deteriorating the qualifications of the Fellows; but surely some further concession might be made to the claims of men who, since their retirement from the schools, have been actively engaged in the practice of their profession. We think it would be far from unreasonable that the Member who could adduce evidence that he had been actively engaged in practice since the date of his diploma, might be allowed to present himself for examination after *eight* years, instead of twelve, provided he was not less than thirty years of age; and if the requirement of a "competent knowledge of Greek" were abandoned in his case, we do not think that either the interests of the public or the dignity of the Fellowship would materially suffer. Were the Board of Health in existence, and we had access to the ear of its members, we would venture to suggest a veto on the bye-laws that went beyond the point now indicated.

2. Although not formally stated in the Bill, it is implied that the College of Physicians shall, in future, have the power of conferring the degree of M.D. on any practitioner who "shall have attained the age of forty years, and shall have been examined by the Royal College," and such graduate "shall be entitled to be registered by the Council as a Physician." This regulation has been made with the express object of enabling general practitioners—who have not, in early life, had the opportunity of taking an University degree, but who, from circumstances, may be extremely well qualified to practise as physicians, and are desirous of doing so—to join the incorporation of Physicians. It is a medium of which we need not doubt that many will avail themselves, since we have always seen the ranks of the physicians plentifully supplied with most respectable members from this source under the old system, when University degrees were more easily attainable—or, at least, when they were more valid—than they will be in future. Still, it must be admitted, that the proportion of Licentiates who will seek this form of incorporation will be very small; and when the advanced period of life at which it is available is considered, it can, in fairness, be regarded less in the light of a corporate privilege conferred on the body of general practitioners than as a graceful means of benefiting and honouring individual members. Nevertheless, it is but just to consider it as a door, however small, whereby access to corporate rights is, for the first time, legally attainable by this class of the profession.

We have now stated everything in the way of incorporation which the Bill and the Charters provide for the class of general practitioners. Although the provisions are much more liberal than before, and although we believe them to be considerably greater and more comprehensive than has been generally admitted in the late discussions on the Bill, we must admit that they fall very far short of the measure of a complete or fair incorporation. A small number will enroll themselves as Associates or Fellows of the College of Physicians; a large number will join the College of Surgeons as Fellows; yet, we believe, that the majority will still remain as general practitioners, either as Licentiates simply, or as Licentiates and Members of the College of Surgeons. Even of those who join the College of Surgeons as Fellows, a certain proportion will feel that a considerable number of their new colleagues, and all the members of the executive and ruling body, have some interests and some sympathies more or less apart from their own, and probably some prejudices directed towards themselves and their branch of the profession. They will know and feel, also, that so long as they attach themselves to their own branch of the profession, they cannot attain any of the higher offices or honours in the corporation.

These and other considerations, which could easily be adduced, prove plainly enough to us that the incorporation of the Licentiates with the College of Surgeons is, to say the least, defective in many essential particulars. The source of the defects is equally clear: it is that which we stated at the beginning of this section—the attempt to reconcile the tripartite state of the profession of the present day with the bipartite arrangements of its pristine condition. In one word, the College of Surgeons is a College for Surgery and Surgeons; while the General Practitioners are neither physicians nor surgeons, or rather are both, and require allocation in an institution possessing, as a fundamental element, this double professional character. We do not wish to disguise our opinion that more might have been done by the old establishments to meet the altered state of things; but, we believe, that an arrangement calculated to meet the wants of the whole profession could only have been effected by a complete reorganization of the Colleges. And although we are far from thinking this undesirable or even impracticable,—if the Colleges could be induced to take an enlarged view of their own interests and the interests of the profession generally,—we do not feel ourselves called upon to discuss matters which can have no practical issue at the present time. The actual question before us is the incorporation of the general practitioners, under existing circumstances.

After much consideration, we have come to the conclusion that it will be for the benefit of the profession generally, that, in addition to the existing medical corporations, a third should be established for the class of general practitioners. In advocating such a measure, however, we are influenced by a very different spirit from that which seems to have suggested the same idea to other writers and speakers on the subject. Our object is to unite the different classes of the profession more closely together, not to separate them. We wish all who agree with us in thinking that medicine is one and indivisible, would also agree with us in endeavouring to make its professors equally united.

Objecting as we do, for reasons detailed in a preceding section of this paper, to the enjoyment by the Colleges of Physicians and Surgeons, of any direct participation in the government of the profession as a body, or to the exercise by them of any authority in testing the qualifications of Licentiates, it may be supposed that we would be far from according such powers to the new incorporation. In our view of the case, the rights, privileges, and duties of a medical college or incorporation, should have exclusive reference to its own members, as members of the incorporation, not as members of the medical body at large. The profession, as a body, must be constituted, governed and regulated by the State, or by an authority created for this purpose by the State; in the present case by the Council of Health and Education. It will be quite enough for a College to govern and regulate itself in its corporate capacity, and to superintend the scientific establishments which must always form an essential and important part of its constitution.

Founded and chartered on these principles, we think that a new College, under the name of "The Royal College of Medicine and Surgery," or under any other appropriate designation, would, as we have already said, be a decided benefit to the class of general practitioners, and to the profession generally. We have not here space, nor is it our province, to enter into particulars respecting these benefits, or respecting the organization of the new institution. Many of its benefits are, indeed, too obvious to require illustration; they are almost all comprised in the very term incorporation. Such a College, would of course, as the acknowledged representative, head, or, if we may so speak, impersonation of the class, stand in the same relation to its own members and the government, as the other incorporations do. It would have a right to be consulted by the Board of Health on the interests of its members, and it would have a right to lay its views, its wishes, its opinions, its grievances, in like manner, before the governing body. It was entirely from the want of some such institution as this, that in the constitution of the Council of Health as proposed in the Bill, no members belonging to the class of general practitioners were nominated. The omission was not an intentional oversight, but the necessary result of the non-existence of any body representative of the class in question.

The proposed College would be no less useful in what may be called its intrinsic influence, or in its relations to its own members. By its Bye-laws it could regulate many important particulars as to professional proceedings, which are now left entirely to the discretion of individuals, to the manifest injury of the professional character. Having the power of excluding and detruding as well as of admitting members, on fixed conditions, it would form a court of appeal in all cases of doubt or difficulty, and would be a tribunal to judge and adjudicate in matters which, though beyond the pale of the laws of the land, might yet be of vital importance to the character of individuals or of the profession.*

An important feature of the College would, of course, be its scientific and literary department. It must possess a house, at least, with a library and theatre. A museum would hardly be required while the Hunterian

* As an illustration of what might be done in this way, we may here notice two cases —one of *exclusion*, one of *detrusion*—which might fairly come within the jurisdiction of the College, although not amenable to the law of the land. 1. Every one admits that the vendors of secret remedies, and advertising quacks are a dishonour to any incorporation: it might be a rule of the new College tha any of its members or fellows, proved to be such, should be instantly struck off the list. 2. Many are of opinion that it is very desirable to discountenance the keeping of open shops for the sale of drugs, &c., after the fashion of chemists, as a practice derogatory to the character of general practitioners: it might be made a rule, that the proprietors of such shops should not be admissible as members of the College.—We know very well that such exclusion or detrusion by the College could not entirely suppress the practices complained of; but there can be no doubt that it would have a great moral effect in putting an end to them, provided the College preserved such a character as made affiliation with it an honour, and consequently an object of desire.

was still incomplete; or, if founded, it would be as a succedaneum to this magnificent national collection. Our views as to the future relations of the old colleges with the new, would be sadly disappointed, if there existed between them any other than a friendly spirit of mutual accommodation and generous rivalry in scientific progress. The new College would have its scientific as well as its business meetings; and in organizing the literary and scientific part of the scheme, it would be an easy task to afford facilities for carrying its labours in this direction beyond what has been hitherto effected by the old Colleges. So long as it was governed by the proper spirit, it could not fail to advance the best interests of medical science at the same time that it conciliated those of its individual members.

The constitution of such an institution would of course be framed on a liberal basis, and its government be thoroughly representative. But, we trust, that its founders would guard against the danger of making it too democratical. We would suggest that every Licentiate should be immediately admissible as MEMBER without ballot, on the payment of a small entrance fee; that he should continue as MEMBER for five years, with enjoyment of all the other rights and privileges, except that of voting or of being eligible to office. At the end of five years, the members should have the right, from seniority alone, to claim to be admitted as FELLOWS under restrictions similar to those we have proposed in the case of the College of Physicians, (p. 206.) Another small fee should be paid for the Fellowship which would of course entitle its holders to the highest privileges of the College.

According to our views, then, as above detailed, the proper organization or incorporation of the profession in England would be as follows:

I. A general Governing Board (THE COUNCIL OF HEALTH AND MEDICAL EDUCATION), having the exclusive regulation of the education, mode of examination, registration, and licensing of medical practitioners of every class.

II. AN EXAMINATION BOARD, appointed by the Council of Health and unconnected with the Colleges, for testing the qualifications of candidates for the license to practise. No one to be permitted to enter the profession without being examined by this Board, and obtaining from it Letters testimonial to the Council of Health, authorizing the granting of the License to practise as a Licentiate of Medicine and Surgery; and no one, not possessing this License, to have any legal right to practise in any department of the profession.

III. A College of Medicine and Surgery for the [voluntary] incorporation of Licentiates of Medicine and Surgery, to be instituted by Royal charter under the name of THE ROYAL COLLEGE OF MEDICINE AND SURGERY, with power to frame laws for the admission of its members (MEMBERS and FELLOWS), for the government of its affairs, electing officers, &c. &c.

IV. A College of Surgery (THE ROYAL COLLEGE OF SURGEONS OF ENGLAND), with power to frame laws for the admission of its members (MEMBERS and FELLOWS), &c. &c.

V. A College of Medicine (THE ROYAL COLLEGE OF PHYSICIANS OF ENGLAND), with power to frame laws for the admission of its members, (ASSOCIATES AND FELLOWS), &c. &c.

### VI. *Of the Bill, in relation to the Representation of the Profession.*

It will be understood from all that we have now stated on the subject of Incorporation, that we consider this, if properly carried out, as comprehending all that is needed in the way of Representation. It must not from this be imagined that we disavow the principle of Representation as one that ought to belong to the medical profession; on the contrary, we maintain that it is an essential principle in every arrangement devised for its organization and government. The only difference, we think, that can exist on this subject among men of understanding and liberal views, regards the amount of this representation and the manner in which it is to be carried out. It must exist somewhere. Our opinion is very decided that it ought to exist in the Colleges or Incorporations; but if it is not allowed to exist there, it must find a place elsewhere. We are quite clear, however, that the Council of Health and Education, as proposed to be established by the Bill, ought *not* to be a body representative of the profession. Its duties are very different from those properly belonging to a representative body. It has the mixed functions of a judicial and executive body. It ought not to represent the profession; but it ought to be, and must be a court open to the representations of the profession. It must be authorized and obliged to consider and to decide upon every question of importance brought before it by authorities acknowledged by the profession: it will have to carry into effect every measure concerning the profession which, either by direct legislation or by legal inference, comes fairly within its jurisdiction.

With the single exception of Dr. Davies, no one of the numerous writers and still more numerous speakers on this subject, seems to us to have taken the proper view of the question. Convinced as they all properly are of the necessity of representation, they have all, in our judgment, made a mistake as to its true character and proper allocation. Having been so long accustomed to see Colleges and Representation disconnected, they seem to have imagined the two things to be incompatible, or, at least, practically unsusceptible of union. And this view of the case has doubtless been greatly strengthened by the fact of a similar view having been taken and propounded by government. The Council of Health, as proposed to be constituted by the Bill, is a rude attempt at representation. But if attempted to be so carried out, the plan must prove a complete failure. It proposes to combine incompatibilities. By it the profession would be badly governed and worse represented. By cheating with the semblance it would divert attention from the substance. The only true and useful representation, of which the medical profession is susceptible, is corporate representation, representation within the Colleges. Dr. Davies's remarks on this point are strictly in accordance with our own views:

"A difference of opinion," he says, "may arise respecting the composition of the 'Council.' It has been urged, that the superintending council ought to be elected by the members of the profession at large; or, as the expression has been, that 'the profession ought to be allowed to govern itself.' Now, in determining this point, we ought to take into consideration the object of the Council, and see what analogy there may be traced between it and any part of our national representative system. It appears from the Bill, that the main object [one of the main objects] of the Council is to compel the different colleges to do their duty; in other words, to see that the education, the examination, and the fees required by the different colleges, shall be *uniform*, as far as uniformity can be carried into effect. From this it follows that the Council is intended to be more of an *executive* than of a *legislative* character; and, from analogy, it may be compared more to the *ministerial* than to the legislative department of the state.................. ......

"Admitting that the elective machinery might work well in the interior economy of the different *colleges*, it does not follow that it would answer the purpose equally well in its application to a *superintending Council* of Education. The principle may apply to the colleges, because they generally possess certain property, which belongs, or is supposed to belong, to the members in common; so that, according to the principle of representation, they ought all to have a voice, indirectly, in the management of the corporation; whereas the Council, under Sir James Graham's Bill, is not intended to hold any property, but is to be paid (if paid at all) by the Lords of the Treasury, who are of course answerable to *Parliament* for their acts." (pp. 70-1.)

All that seems to us necessary for the constitution of such a fair and free representation of the profession as ought to satisfy men of liberal yet practical minds, is comprehended in the following simple arrangements:

1. Such an incorporation of the profession in the colleges as we have above proposed.

2. The right of the incorporations to lay their representations before the Board of Health, either by written petition or remonstrance, or by deputation.

3. The assurance that the representations made are attended to by the Council.

This last most important condition is admirably secured by one of the clauses of the Bill, which, though unostentatiously introduced, is one of the most valuable in it. It is this:

"Be it enacted, that minutes of the proceedings at all meetings of the Council shall be drawn up and fairly entered in books, to be kept for the purpose; and such minutes shall be at all seasonable times open to the inspection of any person appointed for the purpose of inspecting them by any of the said universities and colleges." (§ 12.)

With the power to discuss their particular affairs, as well as what concerns the profession generally, openly and by corporate authority, in the colleges; with the right to lay the result of their discussions before the Council of Health; and with the check on the proceedings of the Council supplied by the admirable clause quoted—a clause which subjects the whole conduct of the Councillors to the control of public opinion—the profession need have no fear as to the administration of their affairs being conducted in accordance with the expressed wishes of the members at large.

### VII. *Of the Bill in relation to the Government of the Profession.—The Council of Health and Medical Education.*

For the first time in this country, an attempt is to be made through the instrumentality of this Bill, to govern the Medical profession on rational principles, or indeed to govern it at all;—for the partial and contradictory rule of the Colleges, heretofore alone prevalent, cannot be regarded as government, in the ordinary sense of the word; or if government, certainly of the kind which the poet tells us prevails where—

> "the ancestors of Nature hold
> Eternal anarchy, amidst the noise
> Of endless wars, and by confusion stand:
> . . . . . . . . Chaos umpire sits,
> And by decision more embroils the fray
> By which he reigns."

It is hardly befitting language to speak of the *importance* of such a measure; its vital *necessity* is so palpable. The profession had long reached such a condition of confusion and misrule, that its regulation was imperative. The measure proposed in the Bill to effect this purpose, is, in principle, precisely such as would naturally suggest itself to every sensible person accustomed to the institutions of a free country. The entire government and supervision of the profession is to devolve on a Council composed chiefly of medical men, and presided over by one of the principal Secretaries of state. Although the only duties of the Council that concern us at present, or, indeed, that are mentioned in the Bill, are those relating to the government of the profession, it is, no doubt, intended that this body should also perform duties of an entirely different, though not of a less important kind;—those, namely, which have reference to the Public Health. Hence its double title—The Council of Health and Medical Education.

It is evidently, and very properly, intended that the Council should have complete control over everything relating to medical education and the qualification of Candidates for the license to practise,—as far, at least, as the unrepealed laws affecting the Universities, Colleges, and Corporations will permit. Many of its duties and powers are specified in the Bill: others are only implied. The following are most of those specified:

1. To superintend the Registration of medical practitioners, and publish a Register of their names.

2. To grant Licenses for Practice on receiving Letters testimonial from the Examining Boards.

3. To regulate the scheme of medical education and fees in the different schools, and render them, as far as practicable, uniform.

4. To have the *veto* on all Bye-laws of the Colleges of Physicians and Surgeons.

5. To enforce the Registration of medical students at the different schools.

6. To settle differences among examining bodies, as to the place, time, or mode of examining; to demand particulars of the examinations; to send their secretary or a member to attend at the examinations; and to refuse the testimonials of bodies whose regulations are deemed insufficient.

7. To specify the institutions which shall be considered *public*, in relation to the appointment of medical officers; and to specify the form of the testimonials required of candidates for such appointments.*

* The power conferred by the last clause (7) will require restriction. We think the right to nominate as officers of hospitals, members ef *any* of the *three* colleges, cannot be taken away from the Governors. But we believe the *result* would still be the same; —the members of the most esteemed class would be preferred as at present.

We fain hope that to the duties of the Council mentioned above, we shall be able to add the following, among others, before the Bill finally passes the House of Commons:

8. To fix the precise amount of preliminary education, which shall qualify for registration as a medical student.

9. To elect the Examiners of the General Examination Board.

10. To institute legal proceedings against persons for practising without a license.

It is manifest, we think, that a body having to perform duties of this nature ought not, as we have already stated, to be a representative body. That it ought to contain among its members individuals thoroughly acquainted with the condition, habits, feelings, wants, wishes, of every class of practitioners in every part of the empire, no one will dispute; and in this sense, it ought certainly to be representative: but this is a very different thing from what is commonly understood by representation, and which has been so loudly and almost unanimously contended for by the writers and public speakers during the discussions which have taken place since the promulgation of the Bill. In these discussions it has been generally maintained that the different incorporations and classes of practitioners should have the exclusive right of selecting and electing certain members from their own body, who should specially represent them and advocate their particular interests in the Council. We think this is a great mistake; and, we believe, that a body formed on such principles would partake more of the character of the Miltonic council quoted in the beginning of this section, than of a calm, deliberative, judicial, executive, impartial, practical board, such as is required for the regulation of a scientific profession. This false view, if not suggested by the government scheme for constituting the Council, has been unquestionably greatly strengthened by it; and, we fear, it will be no easy matter now to convince the profession that they are mistaken.

We may give here one illustration of the evils likely to result from making the Council representative. A complete third of the members is to be furnished by the Colleges of Physicians and Surgeons (3 each); and if the principle of representation is adopted, it is impossible to deny the right of the general practitioners, when incorporated in one or more colleges, to send an equal number at least (3); one half the Council would thus consist of deputies of the colleges, and, doubtless, members of the college councils. Now it is one of the most important duties of the Council of Health to examine and approve of the bye-laws of the colleges, or to put a *veto* on their enaction; and one half of its members—by a thousand accidents converted into a majority—would thus have to decide in the Council of Health on the very laws they themselves had helped to enact, and which, as honest *representatives*, they must now support. It is quite needless to do more than hint at the enormous evils such a species of legislation might lead to. Even with colleges thoroughly representative, it would be singular if the profession at large did not suffer; with the colleges as they exist at present, and as it is at least probable they may exist in future, the interests of the profession would inevitably be sacrificed to the fancied rights and interests of the corporations. No doubt, the clashing of interests of the two or three classes represented might tend to prevent the aggrandisement of any one. But would not this very *clashing* tend, also, to produce either a barren neutrality of action or a corrupt compromise of rights?

Looking, then, to what we conceive to be the proper character of the council and the nature of the duties it will be called on to perform, we are of opinion that its members ought to be chosen by the crown. That the great majority of these members should be medical, and that they should be chosen indifferently from all classes of the profession—physicians, surgeons, general practitioners—we strenuously maintain; but we maintain also, that the grounds on which they should be chosen are not that they are advocates and zealous partisans of their respective classes—nor even that they must necessarily belong to those classes at all—but that they are men of honour, men of judgment, men of sense, men of experience, men of business,—well acquainted, of course, with the special interests of each different department, but regarding these only as forming a part of the interests of the whole profession of which they are the protectors.

We wish we had the power to prevail on Sir James Graham to take this view of the subject, and, disregarding the unwise clamours of the profession on this point, to appoint the members of the council at his own discretion and on his own responsibility. ***But before even we consider him as justified in so doing, he must previously have interposed his authority to make the existing colleges more representative, and have obtained from Her Majesty a charter of incorporation for the general practitioners, based on the most liberal principles.***

We are well aware that many objections have been urged against the expediency of allowing even a portion of the members of the Council of Health to be chosen by the crown; and we are prepared to find our proposition of having the whole so chosen, encountered with a proportionate opposition. After considering these objections with what care we may, we retain our opinion in favour of the government nomination. We do not mean to say that the objections are without foundation, or that the patronage of the crown will be always well bestowed; we merely wish to be understood as stating, that there are difficulties and dangers on both sides, and that we are of opinion that they will be found less numerous and less strong in the case of direct nomination by government than in the case of selection by the colleges and universities. An intermediate plan, however, would equally meet our views, and might obviate some of the objections adverted to. The colleges might be required to return lists of persons deemed by them most fit for councillors (say double or treble the number to be appointed), out of which the minister must make his selection.

The force of the objection founded on the argument that the nomination by the crown annihilates the principle of representation and self-government, so eagerly contended for by the profession, is satisfactorily met, in our opinion, by the counter-arguments, formerly adduced, (1) that such representation and self-government ought to exist, and must exist, in the colleges; and (2) that the proper functions of the Council of Health are incompatible with the existence of such a principle in its formation.

The only other argument of any weight against our view, is one that applies to all government appointments whatever,—the risk that men will be appointed not on account of their fitness for the office, but from other considerations of a political or private nature. We do not deny the chance of this risk. We even believe that it would occasionally be incurred. But are we sure that the choice would always be good if the profession, through the Colleges, had the patronage in their own hands?

We are far from being disposed, on general principles, to enhance the patronage of the crown at the expense of the people; but we confess we should be sorry, for the sake of the people themselves, to see the nomination to public offices transferred from the hands of a minister responsible to the people through Parliament, to the hands of the people themselves, or even to the House of Commons, their organ. And if this holds good with regard to all the other public Boards, and almost all public functionaries, we really cannot see why the Board for the government of the medical profession, should be made an exception. In this case as in the others, we believe that the publicity of the proceedings, and the force of public opinion manifested through its organ the press,—to say nothing of the sense of duty in the mind of the minister—sharpened, it might be, by his consciousness of responsibility to Parliament—would lead, as a general rule, to good appointments. Under any circumstances, it is impossible to believe that so many of them would be bad as seriously to vitiate the efficiency of the Council. And when it is further recollected that the profession, through its organs the corporate bodies, will possess the right to demand the attention of the Council to their representations, and that the Bill gives, in the clause formerly quoted, a direct means of ascertaining publicly, whether these representations have been attended to,—it appears to us that no real grounds exist for apprehending any danger from such a constitution and such an institution of the Council as we are advocating.

But, independently of the question of quasi-representation adopted in the constitution of the Council, as given in the Bill, we think the plan of its formation, as there detailed, is defective in several other respects.

The Council, as everybody knows, is proposed to be composed of eighteen members, viz.

1 President, a Secretary of State: *ex officio.*
5 Regius Professors of five Universities (3 physicians, 2 surgeons): *ex officio.*
3 Physicians: chosen by the Colleges of Physicians.
3 Surgeons: chosen by the Colleges of Surgeons.
6 Other members: chosen by the Government.

The following are some obvious defects involved in this arrangement:

1. If it is meant that all the work is to be done in one place, the Council is much too large for a good working body, and for the duties it will have to perform.

2. It is ill constituted, in respect of the usual places of residence and ordinary duties of its members. Nine of its members reside at a great distance from London; so that regularity of attendance at its meetings would be impracticable on this account alone. Moreover, it is probable that, independently of distance, they would be engaged in other duties which would frequently interfere with their attendance: five, at least, out of the nine, would certainly be in this predicament during one half of the year.

3. Even on the principle of representation, and, indeed, on any principle, the proportion of members given to England is much too small. Of the eleven ex officio and college members, Scotland sends as many as England (4), and Ireland only one less (3); while the actual number of medical men in England is more than five times as great as in Scotland, and more than four times as great as in Ireland.

Strong objections have been urged against the presence of laymen in the Council, but we think on very insufficient grounds. So far from such persons being obstructive to the proper administration of medical affairs, we believe that they would materially promote it. Besides, it seems but fair that, in a matter which concerns the public so vitally, they should have some guarantee that the members of the profession should not have it all their own way. Here we think a sort of representation is very necessary. Class legislation is to be avoided in all cases; and the public have a right to place a check on the class tendencies of the professors of medicine. A strong case in point, in favour of this view of the subject, exists in the Board of Admiralty. It is composed of sea-lords and lay-lords (all appointed by the crown); and it is generally understood that the civil phlegm of the latter is found, on many occasions, to be a most beneficial counterpoise to the professional impetuosity of the former; John Bull being thereby occasionally stinted, no doubt, in the number of his ships, and eke mulcted of his martial glories, yet not without the somewhat satisfactory though very vulgar compensation of feeling his money in his pockets.

### VIII. *Of the Bill in relation to the Professional Privileges and Protection of Medical Practitioners.*

The special privileges and the kinds of protection afforded to the profession by the proposed Bill are chiefly the following:

1. All legally qualified practitioners, as already stated, are to be officially registered, and the register published annually.
2. None but registered persons can hold any public employment, whether in military or civil life.
3. Registered persons shall alone be regarded by the law as medical practitioners, and can alone perform acts required by law to be performed by such,—as the giving of certificates, for instance.
4. Registered persons alone shall be exempt from serving on juries, inquests, &c.
5. Registered persons can alone prosecute and recover payment for medical attendance.
6. Unregistered persons acting in a public capacity as medical practitioners, shall be fined £20 for each offence.
7. Unregistered persons pretending to be registered, or taking the name of Physician, Surgeon, or Licentiate, shall be punished by fine and imprisonment.

These privileges on the one hand, and penalties on the other, are more or less new boons conferred on the medical profession. Taken altogether, they confer upon all its legitimate members a definite and distinctive character in the social body which they did not possess before. By them a broad line is drawn, for the first time, between qualified and unqualified practitioners; between those who, if not certainly learned and skilful, must have been all, at least, trained to medical knowledge and skill, and those who may not have been so trained, and who in all probability will not have been so trained.

These privileges and penalties, it is admitted, would of themselves, suffice to establish the social and professional position of licensed medical practitioners, regarded as individuals, on high grounds; but it is contended that, without some additional aid, they would entirely fail, (1) to secure to them their legitimate rights as members of a learned body recognized by the state; (2) to preserve to the medical profession, considered as a whole, that high and honorable character which ought to belong to it; and (3) to afford to the public at large, any sufficient guarantee that all those permitted by the State to treat their diseases, possess the requisite amount of knowledge and skill.

If, in addition to the excellent enactments quoted, there had been one more, limiting the right of treating diseases in *private houses* as well as in public institutions, to registered practitioners; nothing more could have been desired on the score of protection or privilege. But, without some such clause as this, all the others become comparatively useless; and, under their sole operation, the state of the profession would be, in many respects, in a decidedly worse condition than before.

The grand evil to be apprehended from the want of any restrictive clause in regard to private practice, is, the introduction into the ranks of medical practitioners of a *great number* of men very imperfectly educated, or not educated at all. A few ignorant intruders, here and there, could not materially injure the profession as a body, or make serious inroads on the health of the public at large. But the incapacity of a *large proportion* of the professors of the medical art, must inevitably—and most disastrously—affect the whole profession and the whole community.

The abolition by the present Bill, of the Apothecaries' Act of 1815, which made it unlawful for any person to practise as an apothecary in any part of England or Wales, unless he were licensed by the Court of Examiners, without there being any positive and direct prohibition substituted in its place, leaves the practice of medicine and surgery open to any one who chooses to disregard the indirect penalties imposed by the Bill on those who practise without being licensed. Whoever is unseduced by the flattering prospect of being physician or surgeon to an hospital, dispensary, or workhouse, and is not deterred by the apprehension of having to serve on juries and inquests, or by the incapacity to write a medical certificate on some official occasion, may, without let or hindrance, practise any and every department of medicine and surgery; provided he does not put on his door, or publicly assume, the title of Physician, Surgeon, or Licentiate, and is satisfied with what he can obtain from his patients without suing them in a court of law, for the payment of his bills.

In reference to this most important part of the new Bill, the points of greatest consequence to be considered are the following: *first*, the probability or improbability that persons will enter into private practice, without being licensed; and, if this is thought probable, the extent to which such unlicensed practice is likely to prevail; *secondly*, the nature and degree of evil to the profession and the public, likely to flow from such a state of things; *thirdly*, whether at all, and to what extent the profession and the public have a claim on government to be protected against such evils; and, *lastly*, if so, how this protection can be best obtained.

I. The following considerations, among others, lead us to believe that under the provisions of the present Bill, if carried into a law, the practice of medicine and surgery by unlicensed persons would prevail, and prevail to an extent that would seriously injure the profession and the public.

1. It is well known to all who have had long experience in the profession, more especially in provincial towns and rural districts, that the prohibitory clause of the Apothecaries' Act has had, and continues to have, a powerful effect in preventing and suppressing unlicensed practice. Although rarely put in force, the knowledge that it can be put in force, at any time, and the dread that it may be put in force in any individual case, have a power over intending and actual defaulters, which is notorious. Even the very consciousness of proceeding to act contrary to law, where no special application of it may be expected, operates as a powerful preventive in cases of this kind. In London and four or five other large cities, where the vast amount of population prevents all the members of the professional body from being acquainted with one another, and where it is seldom known or cared for, how or where individual practitioners obtain their living; a man of a bold temperament, and whose conscience is not over sensitive to legal obligations, may very possibly for a long time "drive a handsome business," without possessing any legal qualification. But this cannot be the case in the country, where every one is known to every one, and every one's practice is known, and where the loss of a patient by one practitioner is the gain—and *known* gain—of a patient by another. Here, any drowsy fear of legal consequences, or any little callousness of conscience, on the part of the intruder, is marvellously stimulated by the vivacity of self-interest and the feeling of rivalry on the part of one's neighbours. The consequence is, that the attempted resting-place of the provincial interloper is, generally, to use the vulgar phrase, *made too hot for him*, and he soon ceases altogether in the land, or sets himself down in some remote village, where he starves, without material injury to his qualified brethren—whatever may be the consequence to the lieges. But it is the known existence and possible operation of a restrictive law, in such cases, that supply the whole power by which the suppression of the unqualified practice is effected. If no such law existed, it cannot for a moment be doubted, by men of experience, that a large proportion of such persons would retain the position they had assumed as practitioners.

2. We feel assured that it is a great mistake on the part of the framers of the Bill to believe that the indirect and negative penalties provided by the Bill, would alone prevent, in a very material degree, such persons from practising. We are sorry to be obliged to confess that such a belief assumes the existence of a higher degree of honorable ambition, of just pride, and of a becoming sense of the dignity of the medical office, than exists among the class of persons who would be likely to take advantage of the laches of the law in question.

3. The following are some of the sources whence the abundant supply of irregular practitioners might be fairly expected to flow :

A. *Imperfectly educated medical men from British schools.* It is well known that of the great body of men who frequent the London schools, there is always a considerable number who, from one cause or other, fail to obtain the license of the Apothecaries' Company or the diploma of the College of Surgeons. Even in the present state of the law, a small proportion of these inchoate doctors contrive to worm themselves into practice, under one plea or another: some under the false pretence of being really licensed, some from ignorance of the truth on the part of their neighbours, and others from their good nature or contempt. It is maintained that not a small but a large proportion of such persons would get into practice under the sanction of the new Bill. The class of persons now under consideration contains several genera, which it may be expedient to notice.

*a.* A certain proportion of students, who have commenced their studies with the intention of completing them, and who have prosecuted them with zeal and success, are compelled, from the failure of pecuniary means, to terminate them, at a more or less advanced period, and seek for the means of livelihood within or without the boundaries of the medical profession. Those among them who had been enabled to pursue their studies for a considerable period, and who had thereby attained a fair degree of professional knowledge, seldom leave the profession altogether. Some open shops as chemists, and practise "over the counter;" some venture to practise as general practitioners; but the majority endeavour to get employment as dispensing or visiting assistants to general practitioners. It is a fact well known to the profession, that this class constitutes a large body in England, and contains within its ranks many well-informed and excellent men. Many, however, as may be supposed, are very unfit to be intrusted with medical practice on their own responsibility. It may be fairly presumed that the whole of this class of men, the good and the bad, the well-informed and ignorant, would, sooner or later, become candidates for practice, under the operation of the new Bill.

*b.* A second class, and one, it is to be feared, much more numerous than the preceding, would be made up of those idle and dissipated young men, who, after having spent the full amount of time in the medical schools, are rejected by the examining boards for want of sufficient knowledge. From an authentic table in the Apothecaries' statement, it appears that during the twenty-seven years, extending from 1815 to 1842 inclusive, 11,414 persons presented themselves for examination by the Court of Examiners, and of this number 1502 were rejected. Taking the whole period of twenty-seven years, we have thus an annual average of 55½ rejections; while the average of the last ten years is 76—the increase, no doubt, arising from the increased strictness of the examinations. It is fair to assume that a very considerable number of students of the same class did not present themselves for examination at all, through consciousness of certain rejection awaiting them.

Under the operation of the new law, when it is to be presumed that the examinations would be still more strict, we have a right to expect a still greater number of such failures, and consequently (we assert) a proportionate supply of candidates to swell the ranks of unregistered practitioners. It would surely be according much more to such characters than they have a right to claim, were we to believe that any large number of them would hesitate to commence practice without any license, if they might do so; more especially when it is considered that the pecuniary resources of many of them would not allow them to continue their studies longer, even if they wished to do so.

*c.* A third class of students, we may well believe, encouraged by the example of the two former, would soon spring up, who would frequent the medical schools for a short time, in order to obtain such a smattering of knowledge as might justify to their own tender consciences the attempt to practise, and who would never seek or purpose to seek for an examination of any kind.

*d.* And we think it extremely probable that yet a fourth class of students would be called into existence, who would find schools and teachers, and examinations and titles, more suited to their humble resources and less lofty ambition; and who, after passing a voluntary apprenticeship with their unlicensed fathers or friends, would spend a winter or two in London and return home to treat the lieges, with some new-invented diploma in their pocket, and bearing some obsolete or some modern title which could be displayed on doors and in surgeries, in defiance of section 31 of the new Bill. Need any one doubt, who has known the London grinding system for thirty years, that there would be found men ready and willing to testify to all the world that such students—their pupils—were not only fully qualified to exercise every branch of the profession, but that they were vastly superior to the men who had the legal license and figured on the official register? And would it not be reasonably expected from the pupils and protegees of such persons, that in their localities as "*Doctors*" or "*Surgeon Apothecaries*," they would vaunt themselves in the same strain? And if they did, would it be at all wonderful if a large portion of the public believed them?

A never-failing impulse to the manufacture of the practitioners referred to in the last two paragraphs, would be supplied by the occasional great success of some one of the number, which could not fail to operate on the minds of many parents whose means were insufficient to give their sons an expensive education.

B. *Foreign graduates.* The foreign market also would, doubtless, be called into requisition, as at present, but in a much greater ratio, to furnish its quota to the corps of the unlicensed. Some of the more ambitious youths who could afford it, would go over to Germany, and, after a few months, return with the degree of M.D. in their pocket, from Erlangen or Giessen; while the humbler-minded and the poor would transmit their Testimonials from Dr. M'Crammim and Mr. Grindstone, and their twenty guineas, to the agent in London, and would be dubbed Doctors by the same Universities, by return of post. This class would recruit the ranks both of the general practitioner and the physician, and might be reasonably computed to supply a very considerable number annually. Every one knows that such is the case at present; and it would be indeed strange if the supply were less when the demand was greater.

C. ***Druggists.*** The last stream which we shall here notice as tributary to the great river of empiricism, would have its source in the druggists' shops: and a mighty fine stream (as our friend Dr. Patrick says) would be that same. We believe that within its own channel it would be equal to all the others put together. This hardly needs any illustration; as all who consider the subject, must see at once that such a result is inevitable, if medical practice is left without any legal restriction. Every one knows that a vast number of druggists, even at present, prescribe and practise extensively in their own shops. Many even visit patients at the patients' houses; and it is impossible to doubt that it is the fear of the law and the consciousness of being jealously watched by the licensed practitioners, that prevent them practising much more extensively. If their practice were legalized, as it would be by Sir James Graham's Bill, the whole body of druggists, we verily believe, would rise, en masse, into the ranks of medical practitioners. And assuredly we could not much blame them for so doing. It would be unreasonable to expect that tradesmen should resist a temptation which led so directly and so safely to their own interests; and if the consciousness of incapacity, from want of the proper knowledge, deterred some, it would be an easy matter to find a flattering unction to heal such tender consciences. Within these few years past, thanks to that excellent institution, the Pharmaceutical Society, druggists have received a better and more expensive education in their own proper line. It would be an easy and a natural advance, for such men, under the sanction of the new Bill, to add a course of anatomy and a course of medicine to their pharmaceutical studies; and equally easy, thereupon, to declare themselves ready and willing—over the counter and behind the counter, at home and abroad—to treat all and sundry of her Majesty's lieges who chose to seek their cheap assistance. We should, in a word, have an exact reproduction of the history of the old apothecary developed into a general practitioner; and we feel confident that, before two years should elapse from the passing of the Bill, we should have half the chemists' shops in the kingdom labelled "Chemist and Apothecary," or "Pharmaceutist and Apothecary," instead of "Chemist and Druggist," as at present.

II. We think it impossible, after the exposition now given, to entertain any doubt that, under the Bill as it now stands, the profession would be overrun by an immense number of men altogether unfitted for the practice of medicine and surgery. And it needs no consideration to satisfy every rational mind, that such a state of things would be an enormous evil to the medical profession, and no less a misfortune to the public at large. This proposition is so self-evident, that it would be waste of time to establish it further. It suffices to say, that, in such a state of things, not only the character and standing, but the actual knowledge and usefulness of the whole profession would be lowered and degraded; and the injury to the health of the community would be proportionate to the deterioration of knowledge and skill:

"How," says Mr. Carter, "is the profession to meet with public confidence, if medical men be not distinguished *as a body* for their knowledge and acquirements? If registered and unregistered are to be permitted alike to practise, the *medical character* will be deteriorated. The blunders, ignorance, and misconduct of the unqualified portion will be charged to the *mass:* they will leaven the whole lump. The public will become timid and distrustful of medicine and medical men. Learning and capital will be diverted into other channels."*

III. Although it is almost self-evident that the profession and the public ought to be protected, as far as possible, from the influence of such evils as we have been contemplating, it may not be amiss to make one or two remarks on the subject:

1. The profession seem to have a claim on government for protection against such unlicensed intruders, on the following among other grounds:

*a.* In compliance with the requisitions of the state, as laid down in this very Bill, the members will have to devote many years to the study of their profession, to expend much money, and to undergo strict examinations to test their competency.

*b.* They will have paid considerable fees in order to obtain the license to practise, and to have their names registered as qualified practitioners.

*c.* The privileges exclusively secured to them, in return for such sacrifices—viz. the capability of being appointed to hospitals and other public offices,—are totally incommensurate, so long as the field of private practice is left equally open to those who have made no such sacrifices.

*d.* The members of other professions and the followers of other occupations, who have been obliged to attain a certain amount of knowledge in them—as lawyers, pilots, &c.—are protected against the inroads of the unlicensed; and some sort of protection has been almost universally deemed necessary, by those who have followed any given pursuit, for the sake of distinguishing the competent from the incompetent, and securing a due reward to competency. The national feeling is, and for centuries has been, in favour of the principle of such protection—a point not to be lightly disregarded.

2. The public has a claim on government to be supplied with a body of well-educated medical practitioners, and to be protected against ignorant pretenders to medical skill, on the ground, (*a*) that the practice of medicine concerns directly the health and lives of the community at large, and (*b*) because the community is incapable of judging of the fitness or unfitness of persons to exercise it. It may be said that the public registry of the names of legitimate practitioners supplies the public with the means of judging on this point: but to this it is replied that the practical commentary on the law furnished by the permitted practice of the unregistered, is sufficient to counterbalance the effect of the registry, especially among the great body of the people, who are not likely to know aught of registration or registers. It is no greater interference with the liberty of the subject to say—'Here is a large body of medical practitioners, qualified and licensed to heal disease, choose whichever one among them you please to attend yourself or your family in sickness, but choose not a person neither qualified nor licensed'—than it is to restrict the choice of a client in a suit at law, to the class of attorneys, or a

* Letter to the Secretary of the North of England Medical Asosciation.

captain of a ship, to the class of licensed pilots; and yet no one thinks it would be well to permit unlimited choice as to the individuals who should undertake legal causes, or navigate ships into port. When the welfare of the public demands it, individual liberty of choice and personal freedom of action must alike give way; were it not so, civilization must retrograde, and political liberty itself be extinguished.

"When the liberty of the subject," as Mr. Carter well observes, "threatens to be dangerous to himself, or detrimental to his family or society, it must be restricted. To be consistent, Sir James Graham should not interfere with gambling. Why should people not be permitted to indulge their propensity to be cheated in pocket as well as in health and life? Would the consequences be less injurious to themselves and those who depend on them in the former than in the latter case?"*

IV. Before quitting this part of our subject, it is necessary to notice more particularly, one objection to the restrictive measures we advocate, which has been advanced by many good and enlightened men, and which, we are ready to admit, has always appeared to us to be the only one adduced by the advocates of unrestricted practice, which had any semblance of weight. It is this,—that the limitation of medical practice to men so highly and so expensively educated as the registered class under this Bill will be, would deprive the lowest class of the community—the labouring poor—of the means of obtaining medical relief at all. It is argued that medical practitioners of so high a grade would disdain the scanty offerings of the poor for their learned services, and that this most important class of the community would, in consequence, be neglected in their sickness. It is too true, that even now, the labouring classes in this country are unable to remunerate medical men for their services; how then, it is naturally asked, shall they be able to do so, when a more expensive education warrants, and a more thoroughly exclusive system of practice permits, higher remuneration for services rendered at the bedside of the sick? Is it not the bounden duty of the State to take care that even the risk of such a terrible evil should not be incurred? Is it not better that the whole race of doctors should be ruined, than that such a cruel wrong as this should be perpetrated?

These are most momentous considerations; but we think it can be shown that the course of proceeding we are advocating is far from being inconsistent with their just appreciation.

A. In the first place, we would say, that if the present social and political condition of the people is such as to render it impracticable for the labourer to obtain good advice for himself and family when ill, it is the bounden duty of the state to remove this impracticability.

B. Secondly, we maintain that it would be monstrous if the State were deliberately to meet this difficulty *by lowering the value of the article*, i.e. by supplying the poor with *bad advice* from ill-educated men, and not by raising the ability of the claimant to obtain good.

C. If medical advice were a *mere luxury*, like fine clothes, delicate food, carriages, &c., the State might justly say,—Let a man obtain it of

* Letter to the Secretary of the North of England Medical Association.

the quality he can afford, or go without it; but it is assumed as an axiom that it is *the positive duty* of a State, to take every necessary precaution to preserve the health and life of its subjects; and it is evident that *good medical advice*, in this point of view, comes as much into the category of *necessaries* which the State is bound to place within the reach of all, as food, clothing, housing, &c.

D. It is maintained, therefore, (C) that it is the duty of a government, in the first instance, not only to provide the public with good medical advice, but even to keep *bad* advice out of their reach, as far as this is practicable; and it follows as a corollary (A) that if the actual arrangements of a State prevent any class of its subjects from obtaining this *necessary, these arrangements ought to be changed.*

E. But, even supposing the social and political condition of the poor to remain as at present, and the education and attainments of the general practitioner to be raised by the new Bill—Does it certainly follow that the professional relations between the two will be less advantageous to the former than they are at present? This seems at least doubtful, for the following reasons:

*a*. The present rate of medical charges for attendance on the poor is as high as can well be obtained; therefore the most expensively educated men could get no more, even if they desired it.

*b*. But it is believed that the better the education of practitioners, the more enlightened would be their views, and the more humane their feelings; and that, consequently, taking a truer and kinder view of the actual condition of their poorer brethren, they would be found *cheaper doctors* than even their predecessors had been.*

*c*. The same superiority of intellectual culture would lead to the increased countenance and promotion of the system of *medical clubs* and self-supporting dispensaries, on improved principles; the unhappy opposition to which, by the medical profession, has been mainly the result of ignorance.

*d*. Under any circumstances, (though this alternative we strongly deprecate,) the poor will have the same resource as now, viz. *the chemists' shops*, as we do not believe that what is called *counter-practice* can be prevented by law.

*e*. The more expensive education, under the new system, would diminish the number of persons entering the profession; the members of the profession would, therefore, have a larger sum to be divided amongst them, and could afford to practise at a cheaper rate. For instance, in a certain country district, one licentiate and his family would have to be maintained in place of two; and having double the number of patients, he could afford to charge *the poor*, at least, much less than if his patients were fewer.

* The present practice of the profession corroborates this view of the case. We never see the well-educated and respectable general practitioner oppressing the poor. It is only the unlicensed, uneducated, and disreputable practitioner, who makes heavy charges, and then sues the poor man for payment. The almost invariable practice of respectable general practitioners is to attend the really poor gratuitously, or to see them taken care of in a public institution.

*f.* The apprehension of any place, even the poorest country district, being left without a sufficient medical staff to attend to all the sick, seems altogether groundless. The demand will always create a supply in this as in every other case. Need we seek further evidence of this truth than the notorious fact, that, at this very time, hundreds of Doctors of Medicine are acting as assistants to general practitioners, as surgeons of unions, and as village apothecaries !

V. Having thus, as we think, demonstrated the necessity of protection, it remains to inquire what should be the precise nature and amount of it.

The views of medical men differ a good deal on this point; but it appears to us that the general opinion is in favour of too severe restriction. No one contends that all the forms and colours of unqualified practice, comprehended under the general name of quackery, should be put down, simply because every one admits that this is impossible; but there is a prevalent feeling that the legal restraints should be extended wider than seems easily practicable, or is required by the just vindication of the rights of the qualified by education and law. It seems to us altogether unreasonable that the chemist should be restrained from "prescribing over the counter," as it is called, so far as to be deprived of the power of giving his drugs, simple or compounded, to poor or rich who may call for them, even although he select the medicine, and fix its dose. And if we allow this privilege to the chemist, it seems somewhat difficult to prevent the professed quack from selling his specifics in his own house. For our own parts, although we are fully aware of the amount of evil perpetrated by the race of advertising quacks, both within and without the profession, and although abhorring the cold, calculating robbers and poisoners of the ignorant and confiding, as among the basest of the human race, we do not at present see how these vermin can be effectually exterminated. If any means can be suggested for effecting so desirable a result, without trenching too much on the liberty of the subject, and without endangering the passing of the Bill, we shall embrace them with delight and gratitude. At present, however,—looking at the same time to what will be a reasonable and fair though not a complete protection to the profession, and what will be likely to meet the sanction of the legislature,—we are disposed to urge upon our brethren to be content with the introduction of a clause into the Bill, rendering it penal for all unregistered practitioners *to visit patients at the patients' own houses or at the houses of others.* Such a prohibition, though it would not reach the advertising quacks in their domestic dens, would effectually prevent the introduction of any *large number* of unqualified men into the ranks of the profession. With the exception of London, and a few of the larger cities, no other place could supply the means of livelihood to men forbidden to practise beyond their own threshold. The profession, consequently, as a body, could not be materially injured; and the public could only suffer in that fraction of its mass which now ministers to the cupidity of the public empiric. It is to be remembered, that it is not the class of society that most wants protection, the poor labouring class, who are the prey of these medical arachnidans. They spread their webs only for those who are fat and well-liking, and can bleed; and if the profession generally are not jostled by them, and the poor are safe from their fangs, it may be not unreasonable and possibly may be politic, to leave them for the solace and the patronage of those whose intellectual caliber makes them congenial dupes, and whose worldly means harmonize with their desires.*

---

### ART. XIX.

* The Bill contains several omissions which we hope may be supplied in Committee. We will only advert, at present, to two, and this merely to indicate them, not to discuss them. The first is the omission of all reference to the regulation of the Chemist and Druggist. We think the Board of Health should be empowered to establish a special examination Board for this class, and that no one should be allowed, after a specified time, to open shop, as druggist, without a license obtainable after passing an examination. We think the Pharmaceutical Society might supply the basis and mechanism of such a Board. The second omission relates to the education and licensing of Midwives.

